# Correlation between the fatty infiltration of paraspinal muscles and disc degeneration and the underlying mechanism

**DOI:** 10.1186/s12891-022-05466-8

**Published:** 2022-05-30

**Authors:** Liqiang Shi, Bin Yan, Yucheng Jiao, Zhe Chen, Yuehuan Zheng, Yazhou Lin, Peng Cao

**Affiliations:** 1 Department of Orthopedics, Ruijin Hospital, Shanghai Jiaotong University School of Medicine, 197 Ruijin Er Road, Shanghai, 200025 China; 2 Department of Surgery, Ruijin Hospital, Shanghai Jiaotong University School of Medicine, 197 Ruijin Er Road, Shanghai, 200025 China; 3 Department of Orthopedics, Ruijin Hospital North, Shanghai Jiaotong University School of Medicine, Shanghai, 201800 China

**Keywords:** Paraspinal muscles, Fatty infiltration, Lumbar disc herniation, Lumbar disc degeneration, Inflammation

## Abstract

**Background:**

Low back pain (LBP) is associated with lumbar disc degeneration (LDD) and fatty infiltration of paraspinal muscles. However, there are some controversies about the relationship between LDD and fatty infiltration of paraspinal muscles, and the causation of them is also not clear. Thus, we investigated whether the degree of LDD was associated with fatty infiltration of paraspinal muscles and preliminarily explored the underlying mechanism.

**Methods:**

A retrospective study was conducted on 109 patients with chronic LBP. The degree of LDD was assessed by the Pfirrmann classification. Total muscle cross-sectional area, L4 vertebral body endplate area, and fat cross-sectional area at axial T2-weighted MRI were measured. Multifidus and lumbar disc specimens were taken from eight individuals undergoing discectomy for disc herniation. Gene and protein expression levels of TNF were quantified through qPCR assays and ELISA, respectively.

**Results:**

The relative cross-sectional area, total muscle cross-sectional area, and muscle cross-sectional area asymmetry were not related to LDD. Pfirrmann grades correlated strongly with fatty infiltration of the multifidus and moderately with fatty infiltration of the erector spinae and the psoas muscles. Linear regression analysis suggested that Pfirrmann grades were most associated with fatty infiltration of the multifidus. Compared with II-degree degeneration discs (mild-degeneration group), fatty infiltration of the multifidus in IV-degree degeneration discs (severe-degeneration group) significantly increased, accompanied by increased mRNA expression of TNF. Meanwhile, the protein expression levels of TNF (pg/g protein) in discs (16.62 ± 4.33) and multifidus (13.10 ± 2.76) of the severe-degeneration group were higher than those in the mild-degeneration group (disc: 9.75 ± 2.18; multifidus: 7.84 ± 2.43). However, the mRNA expression of TNF in the multifidus was not significantly different between the two groups.

**Conclusions:**

The results suggest that LDD is associated with fatty infiltration of the multifidus. The possible underlying mechanism is that LDD induces fatty infiltration by inflammation. Furthermore, compared with the erector spinae and the psoas muscles, fatty infiltration of the multifidus shows an optimal correlation with LDD, which may contribute to further understanding of LDD pathology.

**Supplementary Information:**

The online version contains supplementary material available at 10.1186/s12891-022-05466-8.

## Introduction

Low back pain (LBP) is a very common health problem causing global disability, and the economic burden caused by LBP is high compared with other common disorders [1]. As is already known, lumbar disc degeneration (LDD) diseases, including lumbar disc herniation and lumbar spinal stenosis, are the most common diseases manifesting with symptoms of LBP [2, 3]. Unfortunately, the pathological mechanisms of LDD have not been completely understood. Recently, several studies have focused on fatty infiltration of paraspinal muscles in LDD [4–7].

The paraspinal muscles mainly consist of the multifidus [MF], the erector spinae [ES], and the psoas [PS] muscles. These muscles play a key role in maintaining lumbar segment stability and dynamic regulation; they can maintain the posture and restrict excessive intervertebral movement [8, 9]. Previous studies have observed structural remodeling of paraspinal muscles in LDD, including fatty infiltration, asymmetry, and decreased size [2, 10, 11]. Among them, fatty infiltration of paraspinal muscles is closely related to high-intensity pain/disability and structural abnormalities in the lumbar spine [6, 12–15]. However, there are some controversies about the relationship between LDD and fatty infiltration of paraspinal muscles, and the causation of them is also not clear [11].

Recent literature has suggested that there is a relationship between the fatty infiltration of paraspinal muscles and LDD. Disc degeneration and MF fatty atrophy positively correlate at the L3–L4 disc segment in patients with lumbar disc herniation (LDH) [16]. Another study pointed out that the degree of MF fatty atrophy is poorly related to LDD in patients with LBP [17]. Interestingly, one study showed that severe LDD and the fatty infiltration of paraspinal muscles did not display a statistically significant relationship at any lumbar level in women with chronic LBP [18]. Another study reported that patients with severe LDD were more likely to have increased fatty infiltration in the MF and ES [5]. Hence, for solving these controversies above, we need to design and perform a more rigorous and complete experiment to explore these issues.

There are many mechanisms explaining the development of LDD and LBP. It has been shown that inflammation is strongly related to LDD and LBP [19]. Some inflammatory factors can be diagnostic biomarkers of LDD and LBP [19]. Meanwhile, recent research using animal models of spine injury and LDD has revealed the dysregulation of the inflammatory mediators in MF and inflammatory cytokines participated in fatty infiltration of MF [7, 20–22]. In light of previous studies [7, 20–22], the inflammatory reaction might provide a novel insight into the structural MF remodeling after LDD and spine injury. Up to now, it has not been investigated in humans whether inflammation in fatty infiltration of MF increases with the progression of LDD.

The main purpose of this study was to clarify whether fatty infiltration of paraspinal muscles is associated with LDD. We also explored whether gender, age, and body mass index (BMI) correlate with fatty infiltration of paraspinal muscles. Additionally, we intended to preliminarily verify whether inflammation in fatty infiltration of MF increases with the progression of LDD.

## Materials and methods

### Study sample

A retrospective study was conducted on 109 patients with chronic LBP. Between June 2015 and July 2021, a total of 94 patients with lumbar disc herniation (age range, 17–63 years) were included. The inclusion criteria were as follows: 1) single-level lumbar disc herniation at L4/L5 in MRI and CT; 2) radiological examination matching with clinical symptoms (LBP and sciatica); 3) conservative treatment (medical treatment and/or physiotherapy) was ineffective for at least 3 months. The exclusion criteria were as follows: spinal fractures, trauma, spinal cord injuries, spinal infections, spinal tumors, lumbar kyphosis, pregnancy, previous lumbar surgery, scoliosis, spondylolisthesis, comorbidities (diabetes, osteoporosis, uremia, stroke, rheumatoid arthritis, myasthenia gravis, and malignant tumors). Fifteen patients with nonspecific chronic LBP were also selected (Pfirrmann Grade I). The study was approved by the Institutional Review Board of Shanghai Ruijin Hospital.

There were two main reasons for choosing only the L4/L5 segment in this study: 1) the primary site of disc herniations in the lumbar spine occurs at L4/L5 [23]; 2) the L4/L5 disc level plays an important role in the stabilization of the lumbar spine by paraspinal muscles, especially in the early stage of spinal degeneration [18].

### The grading system of LDD

**Fig. 1:**
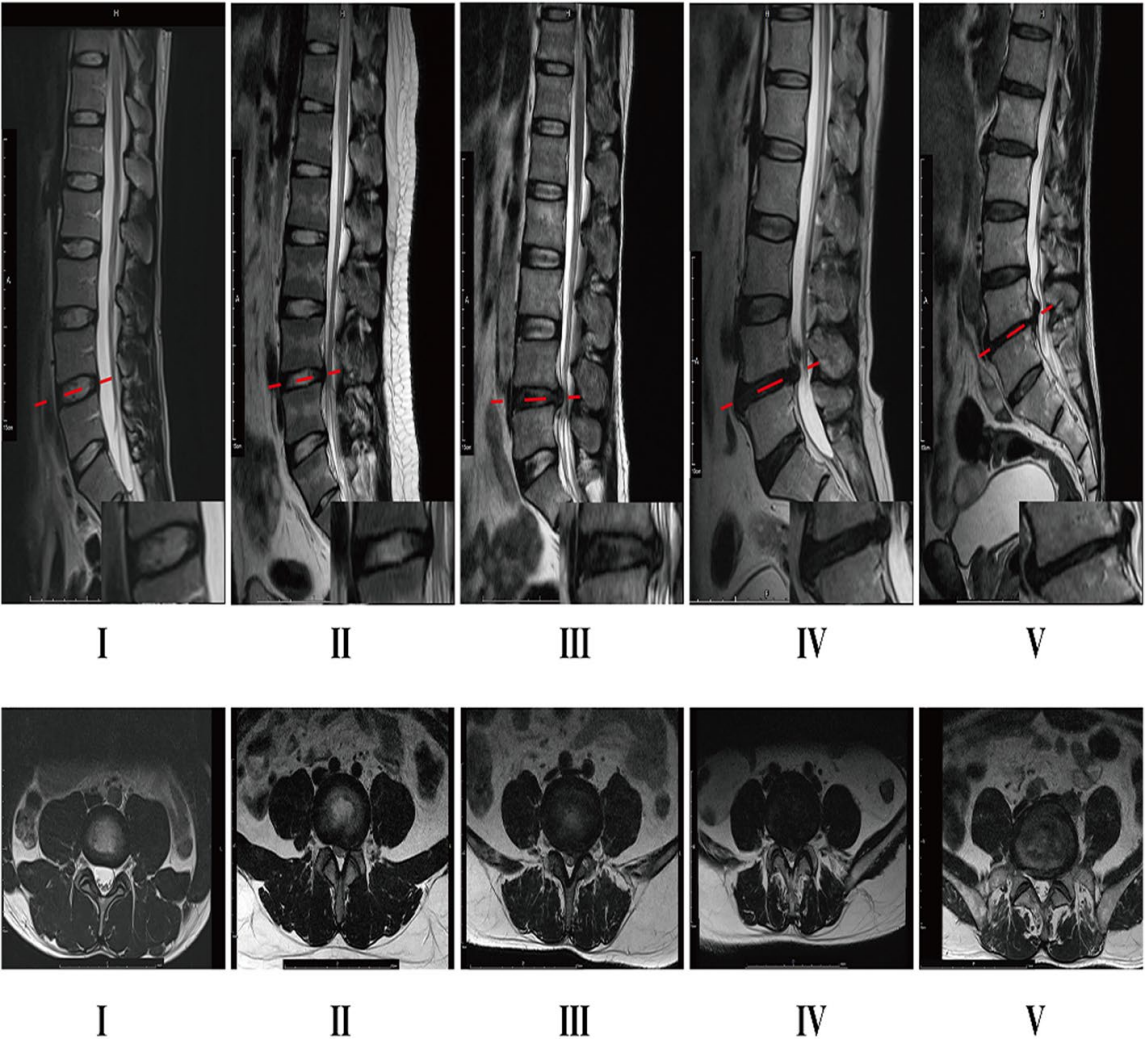
Pfirrmann classification of LDD: Grade I: The structure of the disc is homogeneous, with a bright hyperintense white signal intensity and a normal disc height. Grade II: The structure of the disc is inhomogeneous, with a hyperintense white signal. The distinction between nucleus and anulus is clear, and the disc height is normal, with or without horizontal gray bands. Grade III: The structure of the disc is inhomogeneous, with an intermediate gray signal intensity. The distinction between nucleus and anulus is unclear, and the disc height is normal or slightly decreased. Grade IV: The structure of the disc is inhomogeneous, with a hypointense dark gray signal intensity. The distinction between nucleus and anulus is lost, and the disc height is normal or moderately decreased. Grade V: The structure of the disc is inhomogeneous, with hypointense black signal intensity. The distinction between nucleus and anulus is lost, and the disc space is collapsed. T2-weighted axial images of Pfirrmann Grade of patients

The MRI scans we used were from the Signa HDx 1.5 T MRI system (GE Healthcare, Milwaukee, WI, USA). T2-weighted fast spin-echo (FSE) MRI scans (TR 2550 ms/TE 110 ms; field of view, 320 × 320 mm; matrix, 250 × 324; section thickness, 4 mm; number of excitations, 2) were used to examine the degeneration of the lumbar intervertebral discs and to analyze the fatty infiltration of paraspinal muscles [18, 24–26]. T2-weighted sagittal lumbar spine MRIs were used to evaluate LDD at the L4/L5 disc level by using the Pfirrmann classification (Fig. 1) [27]. Three spine surgeons who were blinded to the clinical data independently assessed the grade of the L4/L5 disc. The intra-class correlation coefficient (ICC) was used to determine the inter/intra-observer agreement using the Pfirrmann grading system. Grade I did not exist in any patients with disc herniation, but it existed in patients with nonspecific chronic LBP. Pfirrmann grades of the patients and their corresponding T2-weighted axial images are shown in Fig. 1.

### Image assessment before surgery

**Fig. 2:**
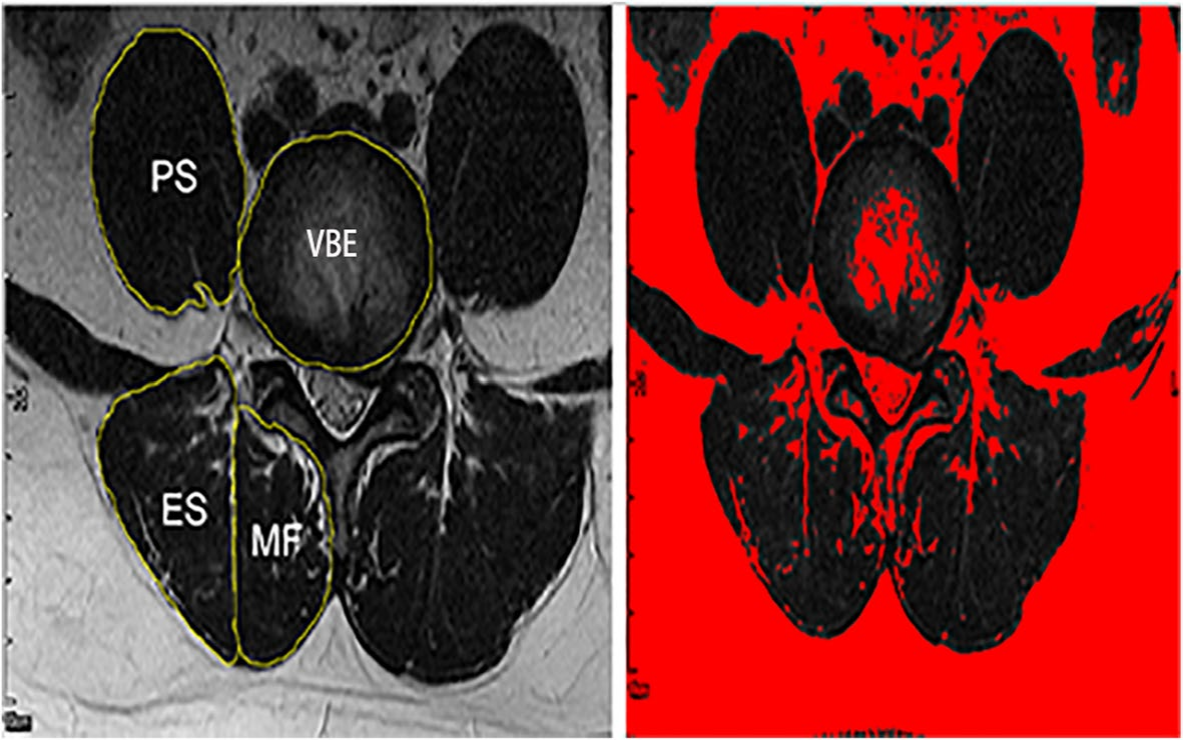
Axial T2-weighted MRI was used to measure the total muscle CSA and fat infiltration rate of the paraspinal muscles at the intervertebral disc level bilaterally. MF: Multifidus ES: Erector Spinae PS: Psoas VBE: vertebral body endplate. The red region of the muscular compartment represents fat

The T2-weighted axial images we collected were used to measure the cross-sectional area (CSA) and fatty infiltration of the paraspinal muscles at the L4-L5 disc level. Muscles were segmented on the axial slice at the L4-L5 disc by locating lines on sagittal images based on previous studies [28–30]. The L5-S1 level was excluded because the axial-cutting gantry was obstructed by the iliac crest and the muscular anatomy is different from that of the upper level [30]. Moreover, the inclination of L5 is often so large that the CSA of paraspinal muscles cannot be appropriately visualized [31]. Quantitative measurement of the paraspinal muscles was obtained from axial T2-weighted images using Image J software (version 1.52, National Institutes of Health, Bethesda, Maryland, US). According to previous studies [2, 25, 26, 30], we manually defined the regions of interest (ROI) per slice for the bilateral paraspinal musculature (Fig. 2). The posterior layer of the thoracolumbar fascia was used as the border of the ES. Measurements were obtained by using a highly reliable thresholding technique, which is based on the difference in signal intensity between the muscle and fat tissue. The fat infiltrated area was measured using the pseudocoloring technique through Image J. In this technique, bright pixels of fat tissue were colored red (Fig. 2). Subsequently, the area of the red region in the muscle compartment was calculated based on the scale of the image. The muscle measurements of interest were as follows: total muscle CSA (TMCSA) and fat cross-sectional area (FCSA). The ratio of FCSA to TMCSA (FCSA/TMCSA) was calculated as an indicator of muscle composition (fatty infiltration). To compensate for the influence of body shape and size on paraspinal muscle CSA, the relative CSA (RCSA) was calculated by dividing the muscle CSA by the CSA of the L4 vertebral body endplate (VBE) at the inferior level. The relative percentage of asymmetry in CSA was calculated using the following formula: [(L – S)/L)] × 100, where L is the larger side and S is the smaller side [25]. The high reliability of the paraspinal muscle thresholding technique of ImageJ software was already validated [32]. All measurements were obtained by two independent spine surgeons with more than 5 years of experience. The ICC was used to assess the inter/intra-observer agreement.

### Clinical symptoms and fatty infiltration of multifidus

From December 2021 to March 2022, 16 patients with L4/5 degenerative disk diseases were selected. We used the visual analogue scale (VAS), Oswestry disability index (ODI), and Japanese Orthopaedic Association Scores (JOA) to evaluate low back pain during hospitalization. According to Kjaer’s study [24], the fatty infiltration of multifidus was rated as grade 0 if normal condition (< 10%); grade 1 for slight fat infiltration (10–50%), and grade 2 for severe fat infiltration (> 50%). Grade 0 did not exist in our patents. The number of patients with Kjaer grade 1 was 11, and the number of patients with Kjaer grade 2 was 5.

### Tissue samples

**Fig. 3:**
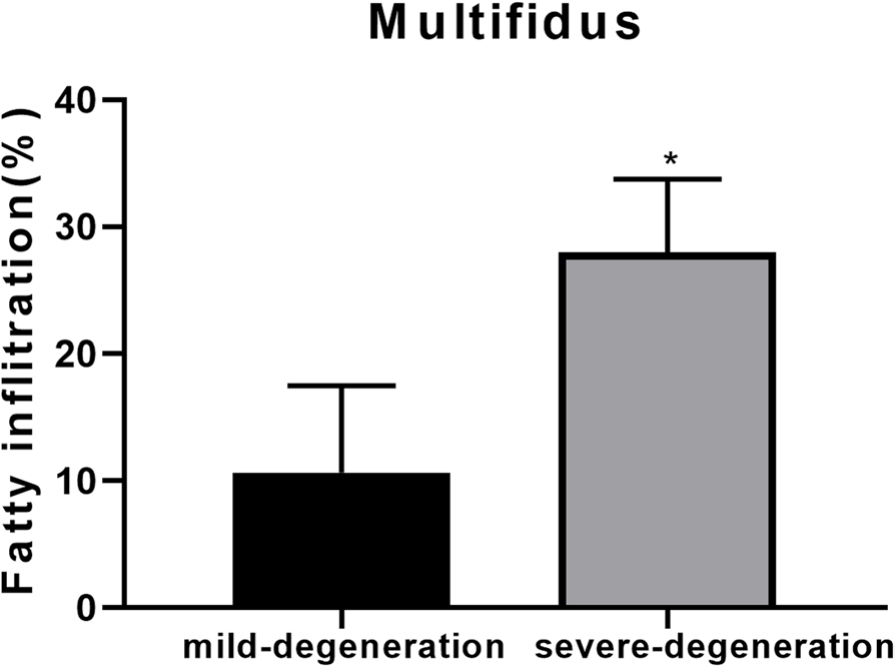
Fatty infiltration of multifidus between mild- and severe-degeneration groups. The fatty infiltration of MF in the severe-degeneration group (Pfirrmann IV) was significantly higher than that in the mild-degeneration group (Pfirrmann II). ^*^P < 0.05

To preliminarily explore whether inflammation was associated with fatty infiltration and disc degeneration, we defined the lumbar disc of Pfirrmann grade II as the mild-degeneration group and the lumbar disc of Pfirrmann grade IV as the severe-degeneration group, the fatty infiltration of MF in Pfirrmann grade IV was significantly higher than that in grade II (Fig. 3). Between May 2021 and July 2021, eight individuals with chronic LBP were recruited for this study. The patients, aged 26–66 years, underwent discectomy for disc herniation. Multifidus and lumbar disc specimens were collected at the time of surgery. All of the patients permitted us to use the discarded tissue for research and signed informed consent forms. There were three patients in the mild-degeneration group and five patients in the severe-degeneration group. There was no significant difference in patients’ characteristics between the two groups.

### Tissue processing

During surgery, the intervertebral disc was obtained from the operated segment. Samples of the multifidus adjacent to the surgical segment were taken from the side ipsilateral to the disc herniation. The samples were washed three times with PBS, quickly placed in liquid nitrogen, and then stored at − 80 °C.

### Quantitative polymerase chain reaction (qPCR)

Total RNA was isolated using TRIzol reagent (Invitrogen, Cat.15596026, USA) in accordance with the manufacturer’s instructions. Complementary DNAs (cDNA) were obtained by using the reverse-transcription kit (Takara, Cat.RR036A, Japan). Subsequently, cDNAs were served as templates, and certain sequences among them were amplified by the qPCR kit with SYBR Green Mastermix (Takara, Cat. RR420A, Japan). The relative expression was calculated as 2^−ΔΔCt^ according to Ct values. The genes and their respective primer pairs are listed in Table 1.

**Table 1:** Primer pairs used for quantitative PCR analysis

Gene name	Forward primer	Reverse primer
TNF	5′AAGGACACCATGAGCACTGAAAGC3′	5′AGGAAGGAGAAGAGGCTGAGGAAC3′
IL1-β	5′GCCAGTGAAATGATGGCTTATT3′	5′AGGAGCACTTCATCTGTTTAGG3′
IL-6	5′CACTGGTCTTTTGGAGTTTGAG3′	5′GGACTTTTGTACTCATCTGCAC3′
IL-8	5′AACTGAGAGTGATTGAGAGTGG3′	5′ATGAATTCTCAGCCCTCTTCAA3′
NOS2	5′ AGGGACAAGCCTACCCCTC 3′	5′ CTCATCTCCCGTCAGTTGGT3′
TGF-β	5′CTGTACATTGACTTCCGCAAG 3′	5′ TGTCCAGGCTCCAAATGTAG3′
GAPDH	5′CTTAGCACCCCTGGCCAAG3′	5′TGGTCATGAGTCCTTCCACG3′

*TNF* Tumor necrosis factor, *IL-1β* Interleukin-1β, *IL-6* Interleukin-6, *IL-8* Interleukin-8, *NOS2* Nitric oxide synthase 2, *TGF-β* Transforming growth factor-β, *GAPDH* Glyceraldehyde-3-phosphate dehydrogenase

### Enzyme-linked immunosorbent assay (ELISA)

Total protein was detected by using enhanced BCA protein assay kit (Beyotime, Cat. P0010S, China). The level of TNF was determined by using the ELISA kit (Beyotime, Cat.PT518, China). All experiment procedures followed the manufacturer’s instructions.

### Statistical analysis

Continuous variables were expressed as the mean ± standard deviation and were analyzed using SPSS 25.0 software (SPSS Inc., Chicago, IL, USA). Categorical variables were compared using Fisher’s exact test. Parametric and non-parametric continuous variables were compared among groups using one-way ANOVA and the Kruskal–Wallis test, respectively. We performed post hoc analysis using the Bonferroni method for continuous variables [33]. The Mann–Whitney U test or independent-samples t-test was also used for comparison between the two groups. To assess the correlation among LDD, age, gender, BMI, and fatty infiltration of paraspinal muscles, we performed Spearman correlation and multivariate linear regression analysis (since the Pfirrmann grading is an ordinal variable). Correlations > 0.7 were considered very strong; 0.5–0.7, strong; 0.3–0.5, moderate; and < 0.3, weak [26]. The ICC was calculated using a two-way random model, characterized by absolute agreement to quantify measurement reliability. Statistical significance was set at α < 0.05.

## Results

### The Pfirrmann classification and demographic details of patients

The inter-observer agreement of the Pfirrmann classification was excellent, with an ICC of 0.974 [0.965 − 0.981], the intra-rater reliability values for the evaluation of discs were also excellent, with an ICC of 0.979 [0.969–0.989], which suggested that the measurement was reliable. We evaluated 109 patients presenting with chronic LBP (mean age, 42.06 ± 12.04 years; duration of symptoms, 30.11 ± 33.97 months;) and L4/L5 disc degeneration grade included grade I (15, 13.7%), grade II (9, 8.3%), grade III (54, 49.5%), grade IV (26, 23.9%), and grade V (5, 4.6%). Further demographic details of the patients with different Pfirrmann grades are shown in Table 2. There was no statistical significance in patients’ characteristics among groups.

**Table 2:** Patients’ characteristics (Data is presented as Mean ± SD)

Demographics	I	II	III	IV	V	*p*-value
Gender(male/female)	6/9	4/5	26/28	12/14	1/4	0.488
Age(years)	35.87 ± 9. 30	39.67 ± 14. 47	41.78 ± 12.45	45.58 ± 11. 20	49.6 ± 6.80	0.073
Height(m)	1.66 ± 0.08	1.68 ± 0.09	1.68 ± 0.07	1.67 ± 0.07	1.62 ± 0.07	0.475
Weight(kg)	64.17 ± 9. 23	68.56 ± 12. 41	70.25 ± 13.12	73.79 ± 14.57	68.10 ± 8.72	0.342
Body mass index	23.11 ± 2.40	24.03 ± 2.13	24.86 ± 4.26	26.24 ± 4.09	25.89 ± 4.10	0.163
(kg*m-2)						
Duration of symptoms(months)	8.13 ± 3.85	10.56 ± 14.08	25.02 ± 30.85	31.62 ± 37.11	39.00 ± 35.62	0.217

### Fatty infiltration of the MF muscle and ES muscle increased with an increase in Pfirrmann grades

**Fig. 4:**
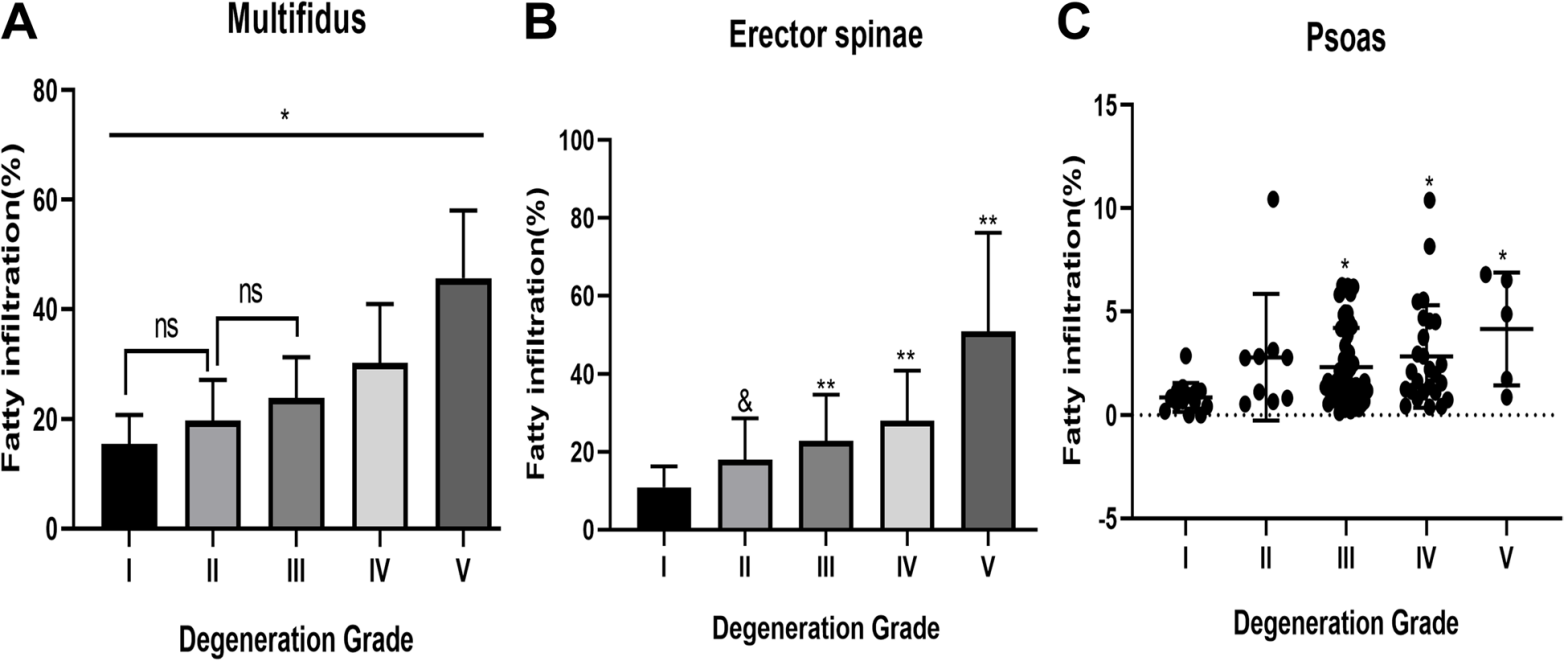
Fatty infiltration of paraspinal muscles among Pfirrmann grades (post hoc analysis with Bonferroni correction). **A** Multifidus The fatty infiltration was significantly increased with an increase in Pfirrmann grades, but Pfirrmann II vs Pfirrmann I、III are not statistically significant. ^*^P-value < 0.05 ns: no significance. **B** Erector spinae Pfirrmann I vs Pfirrmann III、IV、V ^**^P value < 0.01. Pfirrmann II vs Pfirrmann V ^&^*P* value < 0.05. **C** Psoas Pfirrmann I vs Pfirrmann III、IV、V. ^*^Adjust *P*-value < 0.05 (non-normally distributed data)

The inter-observer agreement in assessing musculature were excellent (MF: ICC = 0.989, ES: ICC = 0.990, PS: ICC = 0.934). The intra-rater reliability values for the evaluation of musculature were also excellent (MF: ICC = 0.984, ES: ICC = 0.994, PS: ICC = 0.96). For analyzing the fatty infiltration of paraspinal muscles, we measured the TMCSA and the fatty infiltration of MF, ES, and PS in different Pfirrmann grades. The results showed that the TMCSA, RCSA, and CSA asymmetry of paraspinal muscles among different grades was not significantly different (Table 3). However, with an increase in Pfirrmann grades, the fatty infiltration of MF and ES significantly increased (Fig. 4A, B). Fatty infiltration of PS in Pfirrmann III, IV, and V patients also significantly increased compared with that in Pfirrmann I patients (Fig. 4C).

**Table 3:** Imaging parameters of paraspinal muscles among Pfirrmann grades (Data is presented as Mean ± SD)

Muscle	I	II	III	IV	V	*P*-value
**MF** FCSA/TMCSA(%)	15.44 ± 5.34	19.76 ± 7.41	23.85 ± 7.43	30.30 ± 10.70	45.70 ± 12.31	< 0.001
TMCSA (cm2)	17.27 ± 3.29	16.03 ± 2.82	17.07 ± 3.18	18.25 ± 3.84	16.51 ± 0.77	0.404
VBE (cm2)	13.67 ± 1.88	14.19 ± 2.09	14.88 ± 2.21	14.79 ± 1.85	13.43 ± 4.21	0.103
RCSA	0.64 ± 0.14	0.56 ± 0.09	0.58 ± 0.13	0.62 ± 0.12	0.65 ± 0.14	0.366
CSA asymmetry(%)	8.45 ± 6.22	7.18 ± 5.56	8.22 ± 6.25	8.32 ± 6.59	8.32 ± 6.44	0.998
**ES** FCSA/TMCSA(%)	10.86 ± 5.42	18.07 ± 10.56	22.91 ± 11.84	28.01 ± 12.89	50.79 ± 25.40	< 0.001
TMCSA (cm2)	25.61 ± 6.42	24.46 ± 5.67	22.99 ± 5.41	24.52 ± 6.07	19.82 ± 3.75	0.355
RCSA	0.94 ± 0.24	0.86 ± 0.14	0.78 ± 0.19	0.83 ± 0.2	0.81 ± 0.27	0.099
CSA asymmetry(%)	10.22 ± 6.28	11.88 ± 9.14	11.16 ± 9.18	10.99 ± 9.13	4.79 ± 2.06	0.446
**PS** FCSA/TMCSA(%)	0.85 ± 0.70	2.80 ± 3.06	2.33 ± 1.88	2.83 ± 2.48	4.16 ± 2.72	0.003
TMCSA (cm2)	24.22 ± 6.95	24.72 ± 7.97	24.84 ± 7.99	24.74 ± 9.58	17.88 ± 3.86	0.364
RCSA	0.88 ± 0.19	0.86 ± 0.21	0.84 ± 0.26	0.83 ± 0.25	0.68 ± 0.11	0.618
CSA asymmetry(%)	9.41 ± 5.77	4.56 ± 3.57	7.53 ± 5.81	8.11 ± 6.64	20.30 ± 9.51	0.005

*MF* Multifidus, *ES* Erector Spinae, *PS* Psoas, *VBE* Vertebral body endplate, *TMCSA* Total muscle cross-sectional area, *FCSA* Fat cross-sectional area, *RCSA* Relative cross-sectional area, *FCSA/TMCSA* Ratio of fat cross-sectional area to total muscle cross-sectional area (represents fatty infiltration of paraspinal muscles)

Then, we utilized the Spearman correlation analysis and multivariate linear regression to further confirm the relationship between fatty infiltration of paraspinal muscles and LDD. The results suggested that the Pfirrmann grade had a strong positive correlation with fatty infiltration of MF (Rho = 0.57, *p* < 0.001) and had a moderate positive correlation with fatty infiltration of ES (Rho = 0.49, *p* < 0.001) and PS (Rho = 0.31, *p* < 0.05). Moreover, the multivariate linear regression analysis verified that the Pfirrmann grade, as the first weighting factor, was independently associated with fatty infiltration of MF (β coefficient = 0.515; *p* < 0.0001) and ES (β coefficient = 0.418; *p* < 0.001), and as the second weighting factor in PS (β coefficient = 0.206; *p* < 0.001).

### The correlation between the fatty infiltration of paraspinal muscles and other factors

**Fig. 5:**
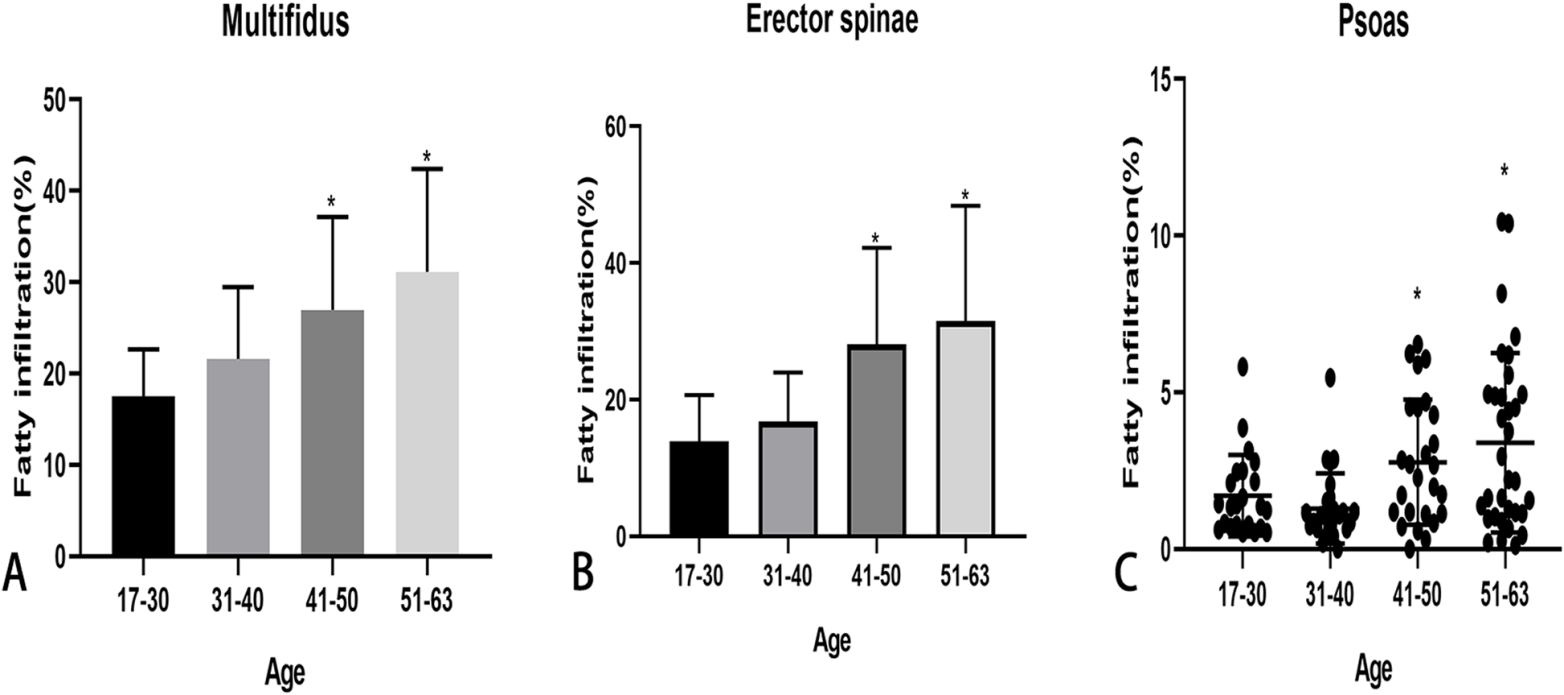
Fatty infiltration of paraspinal muscles among ages (post hoc analysis with Bonferroni correction). **A** Fatty infiltration of Multifidus in subjects aged over 41 years significantly increased compared with that in subjects aged less than 30 years. ^*^P-value < 0.05. **B** Fatty infiltration of Erector spinae in subjects aged over 41 years significantly increased compared with that in subjects aged less than 30 years. ^*^P-value < 0.05. **C** Fatty infiltration of Psoas in subjects aged over 41 years significantly increased compared with that in subjects aged less than 40 years. ^*^Adjust *P*-value < 0.05 (non-normally distributed data)

Consistent with previous findings [5, 24, 26], age showed a strong association with fatty infiltration of the MF (Rho = 0.523, *p* < 0.001) and ES (Rho = 0.512, *p* < 0.001), and a weak association with PS (Rho = 0.287, *p* < 0.05). BMI was not related to fatty infiltration of MF, ES, and PS (Rho =  − 0.027, 0.018, 0.104, respectively; *p* > 0.05). TMCSA of paraspinal muscles in men was larger than that in women, but fatty infiltration in females was higher than that in males, as shown in Table 4. Moreover, the TMCSA of paraspinal muscles decreased with age and fatty infiltration of the paraspinal muscles increased with age, as shown in Table 5 and Fig. 5. The number of each age group was as follows: 17–30 years group (*n* = 23), 31–40 years group (*n* = 27), 41–50 years group (*n* = 26), 51–63 years group (*n* = 33).

**Table 4:** Imaging parameters of paraspinal muscles in gender (Data is presented as Mean ± SD)

Muscle	Male(*n* = 49)	Female(*n* = 60)	*P*-value
**MF** FCSA/TMCSA(%)	21.98 ± 9.09	27.28 ± 10.94	0.02
TMCSA (cm2)	18.34 ± 2.86	16.39 ± 3.38	0.001
VBE (cm2)	15.97 ± 1.69	13.42 ± 1.90	< 0.001
RCSA	0.58 ± 0.10	0.62 ± 0.14	0.599
CSA asymmetry(%)	9.47 ± 7.05	7.16 ± 5.20	0.147
**ES** FCSA/TMCSA(%)	18.63 ± 12.91	27.20 ± 14.51	< 0.001
TMCSA (cm2)	26.19 ± 5.85	21.66 ± 4.78	< 0.001
RCSA	0.83 ± 0.21	0.82 ± 0.21	0.799
CSA asymmetry(%)	10.28 ± 7.64	11.15 ± 9.36	0.978
**PS** FCSA/TMCSA(%)	1.96 ± 1.60	2.71 ± 2.51	0.228
TMCSA (cm2)	30.94 ± 6.89	19.10 ± 4.26	< 0.001
RCSA	0.98 ± 0.24	0.72 ± 0.17	< 0.001
CSA asymmetry(%)	7.75 ± 5.75	8.69 ± 7.26	0.742

*MF* Multifidus, *ES* Erector Spinae, *PS* Psoas, *VBE* Vertebral body endplate, *TMCSA* Total muscle cross-sectional area, *FCSA* Fat cross-sectional area, *RCSA* Relative cross-sectional area, *FCSA/TMCSA* Ratio of fat cross-sectional area to total muscle cross-sectional area (represents fatty infiltration of paraspinal muscles)

**Table 5:** Imaging parameters of paraspinal muscles among different ages (Data is presented as Mean ± SD)

Muscle	17–30	31–40	41–50	51–63	*P*-value
MF FCSA/TMCSA(%)	17.52 ± 5.10	21.58 ± 7.90	26.97 ± 10.16	31.13 ± 11.28	< 0.001
TMCSA (cm2)	18.31 ± 3.02	17.80 ± 4.33	16.57 ± 3.09	16.66 ± 2.40	0.182
VBE(cm2)	14.38 ± 1.81	14.10 ± 2.15	14.67 ± 2.41	15.01 ± 2.34	0.634
RCSA	0.64 ± 0.094	0.64 ± 0.15	0.58 ± 0.12	0.57 ± 0.11	0.038
CSA asymmetry(%)	9.00 ± 6.14	7.84 ± 6.93	7.55 ± 5.42	8.43 ± 6.32	0.058
ES FCSA/TMCSA(%)	13.90 ± 6.79	16.84 ± 7.13	28.13 ± 14.08	31.49 ± 16.89	< 0.001
TMCSA (cm2)	24.73 ± 6.41	24.00 ± 6.16	24.62 ± 4.83	21.99 ± 5.37	0.063
RCSA	0.86 ± 0.20	0.86 ± 0.22	0.86 ± 0.19	0.75 ± 0.20	0.961
CSA asymmetry(%)	12.32 ± 10.03	12.74 ± 10.81	10.42 ± 6.94	8.30 ± 6.04	0.553
PS FCSA/TMCSA(%)	1.70 ± 1.30	1.30 ± 1.12	2.77 ± 1.99	3.39 ± 2.86	0.002
TMCSA (cm2)	29.91 ± 9.79	25.53 ± 8.39	23.31 ± 6.63	20.50 ± 5.13	0.001
RCSA	1.03 ± 0.30	0.89 ± 0.20	0.79 ± 0.17	0.69 ± 0.15	< 0.001
CSA asymmetry(%)	7.30 ± 6.53	8.29 ± 6.32	9.33 ± 7.90	8.07 ± 5.94	0.794

*MF* Multifidus, *ES* Erector Spinae, *PS* Psoas, *VBE* Vertebral body endplate, *TMCSA* Total muscle cross-sectional area, *FCSA* Fat cross-sectional area, *RCSA* Relative cross-sectional area, *FCSA/TMCSA* Ratio of fat cross-sectional area to total muscle cross-sectional area (represents fatty infiltration of paraspinal muscles)

For further exploring the respective weights of these factors, we performed the multivariate linear regression analysis. We found that gender and age were respectively associated with fatty infiltration of MF, ES, and PS (MF: male sex: β coefficient =  − 0.224; *p* < 0.05; age: β coefficient = 0.316; *p* < 0.001; ES: male sex: β coefficient =  − 0.269; *p* < 0.001; age: β coefficient = 0.354; *p* < 0.001, and PS: male sex: β coefficient =  − 0.156; p = 0.084; age: β coefficient = 0.266; *p* < 0.05). BMI had no relation with fatty infiltration of paraspinal muscles (MF: BMI: β coefficient =  − 0.094, *p* = 0.196, and ES: BMI: β coefficient =  − 0.04, *p* = 0.596, and PS: BMI: β coefficient =  − 0.058, *p* = 0.535). The results of Spearman correlation analysis and multivariate linear regression analysis were summarized in tables as additional files.

The RCSA of men in PS was larger than that of women, but the muscle CSA asymmetry did not show a significant difference between men and women. The RCSA of MF and PS decreased with age; however, the muscle CSA asymmetry did not change with age.

### Clinical symptoms and fatty infiltration of multifidus

**Fig. 6:**
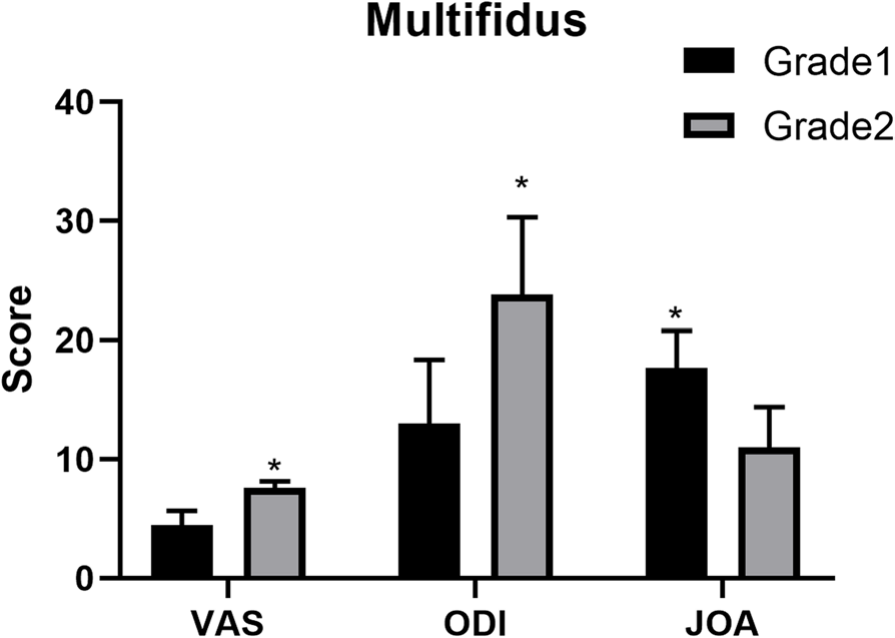
Fatty infiltration of multifidus and pain score. The VAS and ODI in patients with Kjaer grade 2 were significantly higher than those in patients with Kjaer grade 1 ^*^P < 0.05. The JOA in patients with Kjaer grade 1 was significantly higher than that in patients with Kjaer grade 2.^*^P < 0.05

Consistent with previous study [24, 34], we found that the VAS (7.60 ± 0.55) and ODI (23.80 ± 6.50) in Kjaer grade 2 were significantly higher than those in Kjaer grade 1(VAS: 4.45 ± 1.21, ODI: 13.00 ± 5.37), the JOA(17.64 ± 3.14)in Kjaer grade 1 was also significantly higher than that in Kjaer grade 2 (JOA: 11.00 ± 3.40) [Fig. 6]. The results suggested that LBP was associated with fatty infiltration of the multifidus.

### Comparison of inflammatory genes in the lumbar disc between disc degeneration groups

**Fig. 7:**
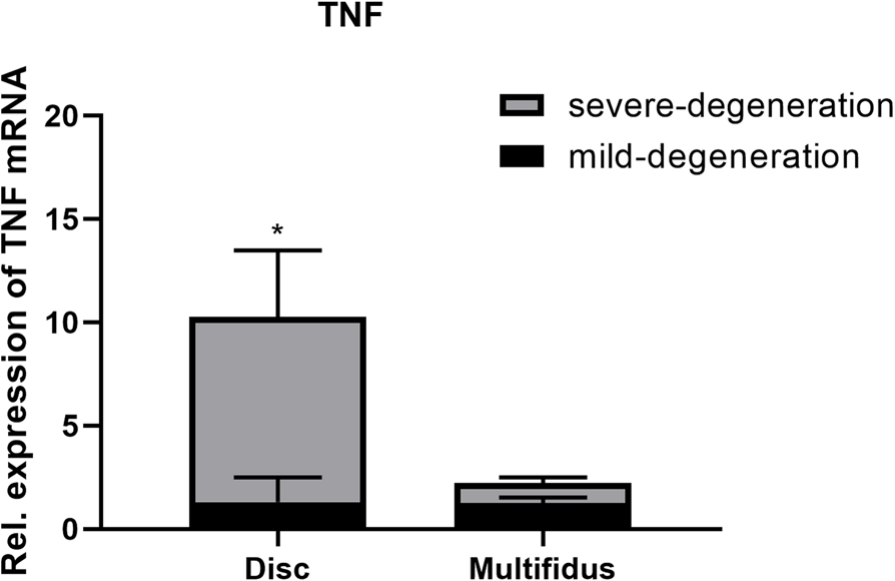
Inflammatory genes in the multifidus and lumbar disc between mild- and severe-degeneration groups. TNF expression of the lumbar disc in the severe-degeneration group was significantly higher than that in the mild-degeneration group.^*^P < 0.05

TNF expression of the lumbar disc in the severe-degeneration group was significantly higher than that in the mild-degeneration group (Fig. 7). There were no differences in the expression levels of IL-1β, IL-6, IL-8, NOS2, and TGF-β between the two groups (Table 6).

**Table 6:** Inflammatory and structural genes in the lumbar disc between mild—and severe-degeneration groups (Data is presented as Mean ± SD)

	Mild-degeneration	Severe-degeneration	*P*-value
Inflammatory markers			
TNF	1.307(1.204)	8.96(3.216)	0.018
IL1-β	0.421(0.501)	0.405(0.217)	0.951
IL-6	0.828(0.471)	0.996(0.734)	0.738
IL-8	0.440(0.501)	0.266(0.180)	0.490
NOS2	3.388 (3.425)	13.950 (8.111)	0.081
TGF-β	2.76 (1.672)	6.943 (2.859)	0.064

Data are expressed as 2^−ΔΔC^.^t^. Mean (standard deviation) is shown *TNF* Tumor necrosis factor, *IL-1β* Interleukin-1β, *IL-6* Interleukin-6, *IL-8* Interleukin-8, *NOS2* Nitric oxide synthase 2, *TGF-β* Transforming growth factor-β, *GAPDH* Glyceraldehyde 3-phosphate dehydrogenase

### Comparison of inflammatory genes in the multifidus between disc degeneration groups

TNF expression of the multifidus was not significantly different between the mild-degeneration group and the severe-degeneration group (Fig. 7). There were also no differences in the expression levels of IL-1β, IL-6, IL-8, NOS2, and TGF-β between the two groups (Table 7).

**Table 7:** Inflammatory and structural genes in the multifidus between mild—and severe-degeneration groups (Data is presented as Mean ± SD)

	mild-degeneration	severe-degeneration	*P*-value
Inflammatory markers			
TNF	1.097(0.563)	1.447(0.339)	0.304
IL1-β	0.621(0.362)	0.827(0.589)	0.609
IL-6	0.468(0.477)	0.818(0.383)	0.293
IL-8	0.407(0.515)	0.462(0.289)	0.850
NOS2	0.815(0.182)	0.972(0.536)	0.649
TGF-β	1.006(0.312)	1.064(0.412)	0.841

Data are expressed as 2^−ΔΔC^.^t^. Mean (standard deviation) is shown *TNF* Tumor necrosis factor, *IL-1β* Interleukin-1β, *IL-6* Interleukin-6, *IL-8* Interleukin-8, *NOS2* Nitric oxide synthase 2, *TGF-β* Transforming growth factor-β, *GAPDH* Glyceraldehyde 3-phosphate dehydrogenase

### Comparison of TNF in the lumbar disc and multifidus between disc degeneration groups

**Fig. 8:**
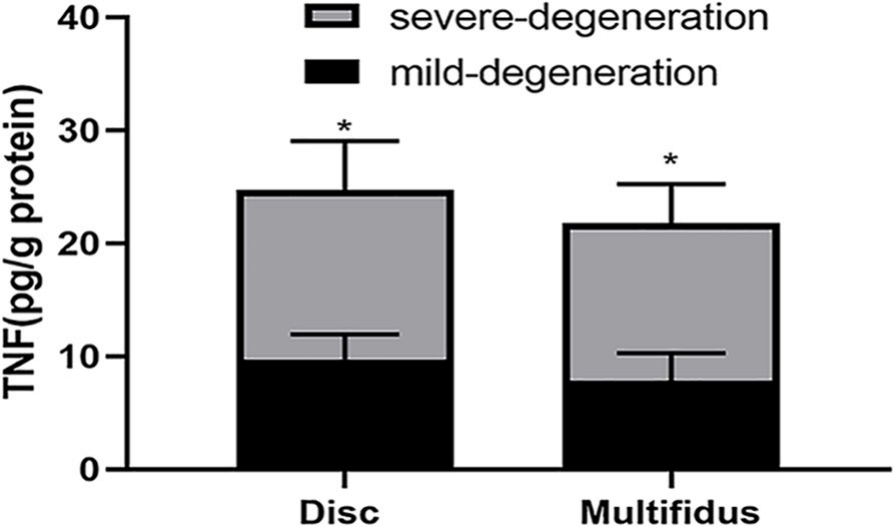
Protein expression of TNF in the lumbar disc and multifidus between mild- and severe-degeneration groups. Expression of TNF of the lumbar disc in severe-degeneration group was significantly higher than that in mild-degeneration group. Expression of TNF of the multifidus in severe-degeneration group was significantly higher than that in mild-degeneration group. ^*^P < 0.05

Based on the result of qPCR, we detected the concentration of TNF in the lumbar disc and multifidus by using ELISA. We found that the protein expression of TNF of the lumbar disc and multifidus in the severe-degeneration group was significantly higher than that in the mild-degeneration group (Fig. 8).

## Discussion

Dysfunction of paraspinal muscles is characterized by fatty infiltration, and it can damage the lumbar spine alignment and impair spinal biomechanics [9, 34]. Recently, an increasing number of studies have focused on the fatty infiltration of the MF; actions of the MF are responsible for more than two-thirds of the stiffness of the spine when in the neutral zone [35] and morphology allows the MF to produce very large forces over a small operating range, which makes the MF ideally suited for stability instead of motion [34]. Hence, the anatomic structure and biomechanics of MF suggest that fatty infiltration of MF may participate in the pathological process of some spinal diseases [2]. Previous studies have found fatty infiltration of MF in LDD [5, 16–18]. However, it is still not clear whether fatty infiltration of paraspinal muscles is associated with LDD.

To verify the problem, we respectively selected patients who had undergone discectomy for severe sciatica and chronic LBP caused by lumbar disc herniation at L4/L5. Meanwhile, we kept consistent baseline demographic characteristics, including age, BMI, and gender, which had not been carried out in previous studies. We also quantitatively detected the rate of fatty infiltration to analyze the correlation between LDD and degeneration of paraspinal muscles, and selected patients with nonspecific chronic LBP as control subjects (Grade I) to better determine the relationship between LDD and fatty infiltration of paraspinal muscles. Moreover, we collected lumbar disc and MF from patients with LDH to explore the underlying mechanism between fatty infiltration of MF and LDD in patients.

First, we measured the TMCSA and RCSA of the paraspinal muscles and found that the TMCSA and RCSA were not significantly different among different degeneration grades. We also evaluate the LBP and fatty infiltration of the multifidus, the results suggested that LBP was associated with fatty infiltration of the multifidus. The results were consistent with some studies [12, 13], the fatty infiltration of paraspinal muscles, but not muscle CSA, was related to high-intensity pain/disability and structural abnormalities in the lumbar spine. A recent study also depicted that fatty infiltration in the multifidus 4 times increased the likelihood of having intense LBP [18]. Ekşi et al. also proposed a new scoring system including fatty infiltration in the paraspinal muscles, they found that scoring (Mo-fi-disc) was correlated with the intensity of LBP [13]. The results showed that the paraspinal muscle CSA may not be associated with LDD. Therefore, some studies suggested that fatty infiltration may be a better indication of muscle degeneration and has a stronger association with physical function than the CSA [13, 26].

Considering earlier studies that had found muscle asymmetry in asymptomatic subjects [36, 37], Hides et al. [38] proposed using 10% or greater asymmetry in multifidus CSA as an indicator of potential spinal abnormality. However, Niemelainen et al. [39] found that paraspinal muscle asymmetry greater than 10% was commonly found in men without a history of LBP, suggesting caution in using level- and side-specific paraspinal muscle asymmetry to identify subjects with LBP and spinal pathology. As shown in our study, the asymmetry in MF CSA and ES CSA did not increase with disc degeneration in chronic LBP, which suggested that muscle CSA asymmetry is not related to LDD.

It has been suggested that marked fat infiltration of MF observed primarily at the level of lower lumbar vertebrae in the general population may be a result of local lumbar pathology, which tends to be most common at those levels [24]. Thus, we calculated the ratio of FCSA to TMCSA (FCSA/TMCSA) to represent fatty infiltration of paraspinal muscles [2, 25, 30]. The results showed that fatty infiltration of MF and ES significantly increased with the increase in Pfirrmann grades. However, significance was weak in fatty infiltration of the PS. Similarly, Parkkola et al. found that patients with chronic LBP had more fatty infiltration of MF and ES but not PS than healthy controls [40]. A recent study evaluated the lumbar spine at all lumbar levels in age-matched women and men with LBP, they also reported that patients with severe LDD were more likely to have increased fatty infiltration in the multifidus and erector spinae muscles [5]. Arbanas et al. suggested that PS remains active regardless of the presence of degenerative and Modic changes of the lumbar spine in patients with LBP [41]. Özcan-Ekşi et al. [18] firstly reported that women with chronic low back pain could have less fat-infiltrated psoas to compensate for more fat-infiltrated multifidus at the L4-L5 disc level. The above evidence suggests a relationship between LDD and fatty infiltration of MF/ES.

To further determine the relationship, we utilized the Spearman correlation analysis and multivariate linear regression. The results showed that the degree of LDD had a strong positive correlation with fatty infiltration of MF and had a moderate positive correlation with fatty infiltration of ES, which confirmed the association between LDD and fatty infiltration of MF/ES. In multivariate linear regression analysis, we found that the degree of LDD was the most important independent correlation factor for fatty infiltration of MF and ES. Nevertheless, we found that LDD was further associated with the fatty infiltration of MF. Based on all the data, we can conclude that fatty infiltration of the MF muscle shows an optimal correlation with LDD, compared with fatty infiltration of the ES and PS.

However, some studies derived different conclusions. These studies suggested that LDD and fatty infiltration of the paraspinal muscles did not have a statistically significant relationship or had a low correlation [17, 18]. Another study reported that patients with severe LDD were more likely to have increased fatty infiltration in the multifidus and erector spinae muscles [5]. In our study, we presented some more powerful evidence than the above studies because we set more stringent inclusion/exclusion criteria, maintained better baseline consistency to reduce data bias, conducted statistical analysis through quantitative determination, and finally drew a conclusion that LDD was closely associated with fatty infiltration of the MF at L4/L5 level.

Currently, the mechanisms accounting for the relationship between fatty infiltration of the MF and spinal diseases are poorly understood. There are many hypotheses regarding the mechanism, such as denervation [42], chronic disuse [43], and inflammation [22]. Especially, inflammation has extensively been studied. In the inflammatory process, TNF is upregulated in LDD; it is regarded as the key mediator of LDD and LBP, as it has powerful proinflammatory activities and is closely related to various pathological LDD processes [44]. Similarly, TNF is also a key regulator of muscle atrophy given that it can induce the upregulation of inflammatory cytokine gene expression and stimulation of the NF-κB pathway in muscle wasting [45]. Moreover, in animal models of lumbar disc injury, greater gene expression of proinflammatory cytokines such as TNF has been found in MF structural remodeling [10, 20, 22]. Interestingly, James et al. [7] found that only the expression of TNF in the MF of patients with high-fat infiltration was higher than that in patients with low-fat infiltration; they further speculated that LDD was related to dysregulation of the inflammatory conditions of the local MF. According to Shahidi et al. [46], high levels of muscle degeneration, inflammation, and decreased vascularity were common in human multifidus biopsies of individuals with lumbar spine pathology in comparison to normative data. However, it has not been investigated in humans whether inflammation in fatty infiltration of MF increases with the progression of LDD.

In this study, we analyzed inflammatory factors in the lumbar disc and MF from patients with LDD. We found that gene expression of TNF of the lumbar disc in the severe-degeneration group (Pfirrmann IV) was significantly higher than that in the mild-degeneration group (Pfirrmann II), but gene expression of TNF in MF did not show a significant difference between the two groups. In contrast, James et al. [7] found that the gene expression of TNF in the MF of patients with high-fat infiltration was higher than that in patients with low-fat infiltration, which may be attributed to their own definition of levels of fat infiltration by using clinical MRI grading scale and the baseline offset existing between the two groups. Moreover, they did not explore the protein expression of TNF. Thus, to further explore whether the protein expression of TNF in the lumbar disc and MF significantly differed between the two groups, we detected the concentration of TNF by ELISA. Surprisingly, we found that the protein expression of TNF of lumbar disc and MF in the severe-degeneration group was significantly higher than that in the mild-degeneration group. In experimental animals [47, 48], TNF was often thought to be produced by the lumbar disc. Moreover, in James’s study [21], their results revealed that spontaneous IDD could cause the dysregulation of the inflammatory pathways active in the multifidus. Because the gene expression of TNF in MF was not significantly different between the two groups. the protein expression of TNF in MF may come from the degenerated intervertebral discs. Based on our results and those previous studies [7, 21, 22, 47, 48], we speculate that LDD is associated with fatty infiltration of the multifidus. LDD may induce the fatty infiltration by TNF. The inflammation, as a core link, may be related to the phenotype. The hypothesis may explain why the fatty infiltration of MF increases with the progression of LDD.

Finally, we continued to analyze the correlation between fatty infiltration of paraspinal muscles and other factors (BMI, gender, and age.). Similar to Kjaer et al. [24], we found that fatty infiltration of paraspinal muscles was not affected by BMI. Another study [49] showed that BMI was associated with severe fatty infiltration in the erector spinae at L2-L3 and L3-L4 levels. But in our study, BMI was not related to fatty infiltration of paraspinal muscles, which may be attributed to our evaluation only at the L4/5 level. Moreover, consistent with previous findings [24, 26, 29, 30, 46], fatty infiltration increased with age, and TMCSA decreased with age. Age was also associated with fatty infiltration of the paraspinal muscles. In terms of gender, fatty infiltration was higher in females than in males, which may be due to their own body composition [24] and our choice of disc level. A recent study [5] comparing age-matched women and men with LBP depicted that women had more fatty infiltration in the multifidus and erector spinae muscles at L4-L5 and L5-S1. Men had more fatty infiltration in the psoas muscle at L5-S1. Patients with severe LDD were more likely to have increased fatty infiltration in the multifidus and erector spinae.

There are some limitations to our study. To avoid the possible interference of other discs, we selected patients with lumbar disc herniation only at L4/L5. Other disc levels were not explored in our study, we will explore them in the future. The tissue samples included in our study were not large; thus, further study needs more samples to further explore mechanisms between fatty infiltration of MF and LDD in humans. It is hard to obtain tissue samples in the pain-free control group of age-matched participants, so animal models of LDD seem more important to observe the fatty infiltration of MF and LDD, and such research will be conducted. Nevertheless, the fatty infiltration and inflammatory cytokine expression were related in our study.

## Conclusions

The study further confirmed that LDD was associated with fatty infiltration of the multifidus. The possible underlying mechanism of fatty infiltration of MF may involve the local inflammatory reaction induced by TNF. However, the TNF in the MF may come from the degenerated intervertebral discs rather than from the muscle itself. Besides, LBP is associated with fatty infiltration of the multifidus. Furthermore, compared with the erector spinae and psoas muscles, fatty infiltration of the multifidus shows an optimal correlation with LDD, which may contribute to further understanding of LDD pathology.

## Supplementary Information

**Additional file 1.**

**Additional file 2.**

## Data Availability

The datasets generated and analyzed during the current study are available from the corresponding author on reasonable request.

## Abbreviations

LDD: Lumbar disc degeneration
TMCSA: Total muscle cross-sectional area
FCSA: Fat cross-sectional area
LBP: Low back pain
MF: Multifidus
ES: Erector spinae
PS: Psoas
BMI: Body mass index
MRI: Magnetic resonance imaging
CT: Computed tomography
FSE: Fast spin-echo
ICC: Intraclass correlation coefficient
CSA: Cross-sectional area
ROI: Regions of interest;
LDH: Lumbar disc herniation
cDNA: Complementary DNAs
ELISA: Enzyme-linked immunosorbent assay
TNF: Tumor necrosis factor
IL-1β: Interleukin-1β
IL-6: Interleukin-6
IL-8: Interleukin-8
NOS2: Nitric oxide synthase 2
TGFβ: Transforming growth factor β
GAPDH: Glyceraldehyde 3-phosphate dehydrogenase
qPCR: Quantitative polymerase chain reaction
VBE: Vertebral body endplate
RCSA: Relative cross-sectional area
