# Supplementary material for: Correlation between the fatty infiltration of paraspinal muscles and disc degeneration and the underlying mechanism
Source: BMC Musculoskelet Disord. 2022 May 30;23:509. doi: 10.1186/s12891-022-05466-8 (PMC9150320; doi:10.1186/s12891-022-05466-8)
Supplement: Supplementary file 1 — Additional file 1. [file 12891_2022_5466_MOESM1_ESM.docx]

Additional file 1: Correlation analysis of fatty infiltration of paraspinal muscles and related factors by Spearman’s Rho test

| fatty infiltration of  paraspinal muscles factor Rho p-value |
| --- |
| MF pfirrmann grade 0.57 < 0.001  age 0.523 < 0.001  BMI −0.027 > 0.05  ES pfirrmann grade 0.49 < 0.001  age 0.512 < 0.001  BMI 0.018 > 0.05  PS pfirrmann grade 0.31 < 0.05  age 0.287 < 0.05  BMI 0.104 > 0.05 |
