# Supplementary material for: Correlation between the fatty infiltration of paraspinal muscles and disc degeneration and the underlying mechanism
Source: BMC Musculoskelet Disord. 2022 May 30;23:509. doi: 10.1186/s12891-022-05466-8 (PMC9150320; doi:10.1186/s12891-022-05466-8)
Supplement: Supplementary file 2 — Additional file 2. [file 12891_2022_5466_MOESM2_ESM.docx]

Additional file 2: Correlation analysis of fatty infiltration of paraspinal muscles and related factors by multivariate linear regression test

| fatty infiltration of  paraspinal muscles factor β-coefficient p-value |
| --- |
| MF pfirrmann grade  0.515 < 0.0001  age 0.316 < 0.001  male sex −0.224 < 0.05  BMI −0.094 0.196  ES pfirrmann grade 0.418 < 0.001  age 0.354 < 0.001  male sex −0.269 < 0.001  BMI −0.04 0.596  PS pfirrmann grade 0.206 < 0.001  age 0.266 < 0.05  male sex −0.156 0.084  BMI −0.058 0.535 |
